# Optimization Design of High-Performance Powder-Spreading Arm for Metal 3D Printers

**DOI:** 10.3390/mi16111194

**Published:** 2025-10-22

**Authors:** Guoqing Zhang, Junxin Li, Xiaoyu Zhou, Yongsheng Zhou, Juanjuan Xie, Yuchao Bai

**Affiliations:** 1 School of Mechanical and Electrical Engineering, Zhoukou Normal University, Zhoukou 466000, China; lijunxin1995@163.com (J.L.); vzhouxy@163.com (X.Z.); zhouyongsheng1@sina.com (Y.Z.); xiejuan119@163.com (J.X.); 2 School of Robotics and Advanced Manufacture, Harbin Institute of Technology, Shenzhen 518052, China; baiyuchao@hit.edu.cn

**Keywords:** 3D printing, powder-spreading arm, topology optimization design, finite element simulation, stress

## Abstract

The powder-laying arm of a metal 3D printer is heavy, which can easily cause long-term damage to the powder-laying servomotor or belt, so it is necessary to design a lightweight powder-laying arm. To this end, we first use 3D modeling Rhino software to rebuild the powder-laying arm, and then, we carry out topology optimization design on the rebuilt powder-laying arm in Altair Inspire software. Finally, we use the Aurora Elva 3D printer to complete manufacturing and assembly to verify compatibility. The results show that the maximum displacement of the original powder-spreading arm is concentrated in the lower right corner at 4.319 × 10^−5^ mm; the maximum stress is concentrated in the middle transition part, decreasing toward the ends; the maximum stress is 3.843 × 10^−2^ MPa; the stress concentration and deformation of the powder-spreading arm when spreading powder is small, which provides a large optimization space. The topology-optimized powder-spreading arm, with a 25% quality objective, maintains the integrity of the connection with the fixing hole while having a large mass reduction. The surface of the parts of the completed 3D-printed powder arm is bright, with low roughness, and there is no obvious warping and deformation or other defects; the completed 3D-printed powder-spreading arm and the assembly of the wall are closely coordinated with each other, and the location of the screw holes is appropriate, having no obvious assembly conflicts between the parts, which lays the foundation for the mass production of the powder-spreading arm of high-performance metal 3D printers.

## 1. Introduction

Three-dimensional printing technology comprises the use of special software for three-dimensional model slicing and layering to obtain cross-sectional data, which are then imported into rapid prototyping equipment using the layer-by-layer material method of manufacturing solid parts. Because of the layer-by-layer method, 3D technology can almost carry out the manufacture of any geometric shape part, with the advantages of processing single pieces, small batches, complex geometric structures, and dense organization of finished parts [1,2,3]. SLM (selective laser melting) molding technology is a 3D printing technology based on the laser melting of metal powder [4,5].

The working process of an SLM printer’s powder-spreading mechanism is as follows: The piston in the powder supply cylinder is driven by a stepping motor, and whenever a layer of the 3D-printed cross-section is finished melting, the piston rises by one layer (the thickness of the rising layer is about two times the thickness of the sliced layer of the processed part) and upwardly ejects a layer of the powder material used for molding; a belt dragged by the motor drives the powder-spreading arm and the powder-spreading scraper to move from the right to the left, spreading the powder on top of the molding piston. Excess powder is pushed into the residual powder recycling bucket, and then, the powder-spreading arm and powder-spreading scraper return to the previous position via left to right movements. At this time, the powder-spreading scraper will scrape the powder that has been spread on the flat powder bed. The effect of the powder-spreading mechanism is related to the quality of 3D-printed parts.

Xiong Jin et al. [6] analyzed the principal components and functions of SLM forming equipment, put forward the overall scheme of a metal powder selective laser melting 3D printing systems based on SLM forming technology, and carried out the real-time control of the laser scanning, preheating, and powder-laying process of SLM motion control systems. Chang Feng et al. [7] analyzed the structure of powder-laying systems and the powder-laying principle of the aluminum alloy constituent laser melting 3D printing equipment independently (developed and produced by Anhui Hengli Additive Manufacturing Technology Co., Ltd.) and tested the powder-laying accuracy of the device, proving that the powder-laying system developed by this company can satisfy the technical powder-laying requirements of aluminum alloy constituent laser melting and molding technology. Shi Zhenwei et al. [8], based on the thickness of the preferred powder, outlined three combinations of process parameters and analyzed the impact of the combination of process parameters on the fundamental properties of selective laser melting technology used to form AlSi10Mg alloy specimens; they determined that a laying powder thickness of 30 μm resulted in specimens with the best surface quality. Yinghua et al. [9] constructed a powder dispenser for delivering different kinds of powder with varying mixing ratios to three to five powder storage tanks; then, through a powder pickup gate and powder channel connected to each tank, the powder in each tank was spread to different areas on the surface of the printing platform. Zhu Tianguang [10] designed a set of high-precision metal powder laser melting zoning 3D printing equipment based on the research and analysis of 3D printing technology. Qi Xiaolong [11] designed metal 3D printer isolation and powder-spreading devices, and modal analyses of the powder-spreading device were conducted, which showed that its first-order intrinsic frequency was close to the vibration source’s frequency. Habiba et al. [12] used the discrete element method simulation tool to study the effect of various input parameters on powder densities and particle distributions during the powder-spreading process. The M250 machine from EOS, Germany, can produce molded parts with close to 100% densities, with dimensional accuracies of 20–80 μm, minimum wall thicknesses of 0.3–0.4 mm, and a stable molding process [13]. The ProX DMP 300 equipment of the U.S. 3D Systems company uses a 500 W laser with a molding size of 250 × 250 × 300 mm, which enables fast mold changes, fast powder recycling, accelerated production speeds, and low material loss rates [14]. To improve the quality of metal 3D printing parts, we previously used the method of mold flow simulation combined with forward design to optimize the structure (deflector plate and air-guide groove) of the gas circulation filtration system of 3D printing equipment. The performance of this method was investigated, and the quality of the molded parts was substantially improved [15,16].

Current research on the powder-spreading mechanism of 3D printers focuses on improving powder feeding and spreading methods, and there is still substantially room for lightweight designs. As the core moving component, the powder-spreading arm, with its traditional heavy design, is prone to high motor loads, fast transmission wear, and poor powder-spreading accuracies, making it challenging to meet the demands of high-end fields. This article investigates the design of a lightweight powder-spreading arm, which addresses exiting gaps, reduce costs, improves efficiency, and contributes to the advancement of SLM technology.

## 2. Materials and Methods

### 2.1. Design Methods

**Figure 1:**
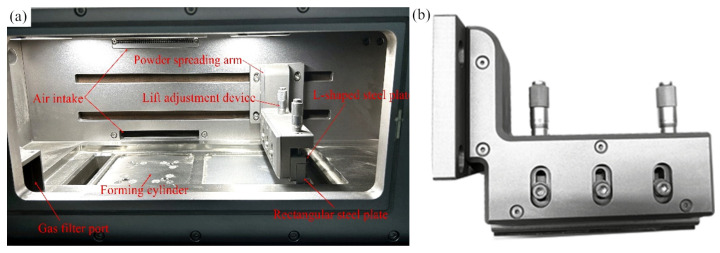
Powder-spreading arm of GYD 150 metal 3D printer: (**a**) internal structure of the molding cavity; (**b**) powder-spreading arm.

The current literature on SLM molding equipment powder-spreading systems and some of the current equipment (such as GYD 150, SLM150, etc.) used in powder-spreading systems are shown in Figure 1. The SLM molding equipment, which comprises a powder-spreading system with a spreading arm installed in the molding cavity (following the method of Lan), often fails to operate effectively, with the arm typically remaining above the powder cylinder on the right. There is an L-shaped steel plate installed under the powder-spreading arm for height adjustments, and the L-shaped steel plate is installed with a rectangular steel plate. A trapezoidal groove is opened on the rectangular steel plate for installing a rubber scraper. At present, the main problem of the powder-spreading system of SLM molding equipment is that the powder-spreading arm is heavy and can easily cause damage to the powder-spreading servomotor or belt after a long period of time. Moreover, the use of the solid powder-spreading arm will also increase the weight of the whole machine and increase manufacturing costs. Therefore, it is necessary to optimize their design. To this end, we use the three-dimensional modeling software Rhino 6 developed by Robert McNeel & Assoc to complete the reconstruction of the powder-spreading arm.

### 2.2. Material and Molding Method

Because the completed powder-spreading arm has a substantial design and is produced as a single piece or in small batches during the product development stage, the use of machining and welding methods significantly increases the cost. Therefore, this paper uses 3D printing methods to manufacture these complex parts. To this end, an industrial high-precision desktop 3D printer (Model: Z-603S), manufactured by Shenzhen Aurora 3D Technology Co., Ltd. (Shenzhen, China), was employed for printing. First, the design of the completed powder-spreading arm was saved in STL format and imported into the 3D printing data processing software JGcreat for printing parameter settings. The specific parameter settings are as follows: the nozzle diameter was set to 1.75 mm; the molding material was PLA; the print layer thickness was set to 0.15 mm; the filler density was set to 18%; the print temperature was set to 203°; the wall thickness was set to 0.6 mm.

The 3D-printed lightweight powder-spreading arm is subjected to surface treatment: first. The support is removed; then, sandpaper is used for rough polishing, and finally, polishing cloth is used for polishing treatment. After the completion of surface treatments, the powder-spreading arm’s parts are assembled to verify their fit and compatibility.

### 2.3. Analysis Methods

First, the Inspire 2023 software developed by Altair was used to carry out finite element analysis on the completed design of the powder-spreading arm; then, topology optimization was carried out in Inspire. The topology optimization of the completed powder-spreading arm was imported into Materialise Magics 22.01 software developed by the Materialise company to remove unnecessary parts; finally, the completed powder-spreading arm was manufactured. Then, the topology-optimized arm was imported into Materialise Magics 22.01 to remove unnecessary parts, and finally, the completed powder-spreading arm parts were assembled and analyzed.

## 3. Results and Discussion

### 3.1. Reconstruction Effect of Metal 3D Printer Powder Arm

**Figure 2:**
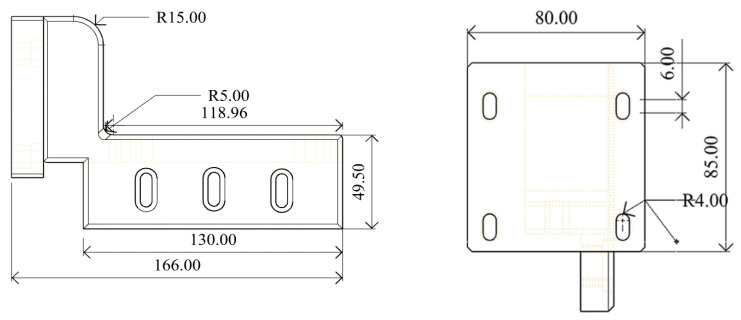
Powder-spreading arm and dimensions of the reconstructed GYD 150 metal 3D printer.

The reconstructed GYD 150 metal 3D printer powder arm in Rhino 6 software is shown in Figure 2, and the dimensions are expressed in mm.

### 3.2. Finite Element Analysis of the Reconstructed Metal 3D Printer Powder-Laying Arm

#### 3.2.1. Load and Constraint Application of the Powder-Spreading Arm

**Figure 3:**
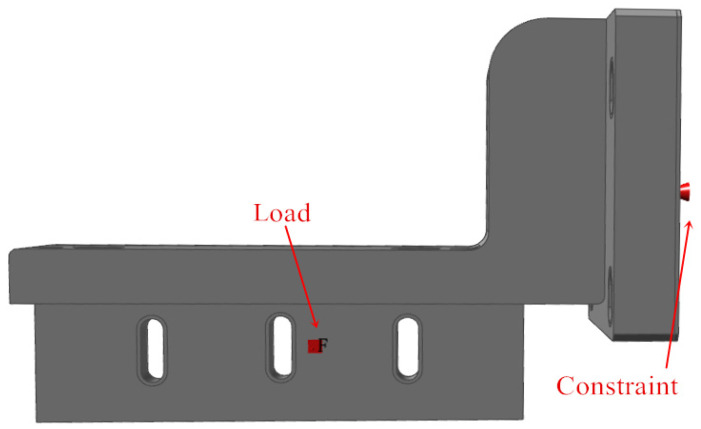
Schematic diagram of load and constraint application of powder-spreading arm.

Import the designed powder-spreading arm into Inspire 2023, and set the unit of analysis as mm kg N s. Set the material of the powder-spreading arm to 316 kg N s. The material of the powder-spreading arm is 316 kg N s. Set the material of the powder-spreading arm to 316 L (density: 8 × 10^−6^ kg/mm^3^; elastic modulus: 0.29; stiffness: 1.95 × 10^5^ MPa). The tap density of 316 L stainless steel powder is about 4.98 g/cm^3^, and the cross-sectional area of the powder cylinder is 120 mm × 120 mm = 1440 mm^2^. When processing 316 L or TC4 powder, the processing layer’s thickness is usually set to 0.25 mm. With three times the powder supply, each scraping and painting operation requires pushing 0.75 mm × 4.98 g/cm^3^ × 1440 mm^2^ = 5.38 g of powder. Therefore, a force of about 0.053 N needs to be applied, and we set a certain margin to apply a force of 2 N at the right end of the powder-spreading arm. The base of the powder-spreading arm is connected to the flange driven by the servomotor such that the base surface can be fixed directly. The loads and constraints applied to the powder-spreading arm are shown in Figure 3. The grid division adopts a triangular grid, with a grid cell size of 6 mm, and the analysis speed is selected more accurately.

#### 3.2.2. Finite Element Analysis Results of Powder-Spreading Arm

**Figure 4:**
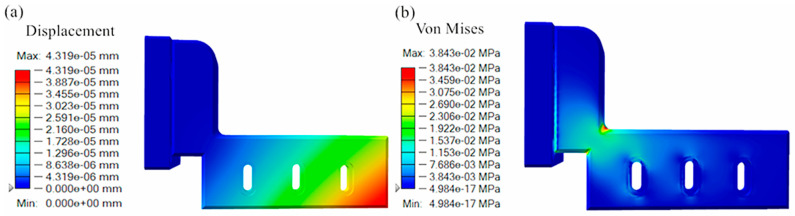
The finite element analysis results of the reconstructed powder-spreading arm: (**a**) displacement cloud map; (**b**) stress cloud map.

The finite element analysis results of the powder-spreading arm are shown in Figure 4. By observing the displacement cloud in Figure 4a, the displacement of the powder-spreading arm from the lower right corner to the upper left corner gradually decreases, in line with the change rule of the displacement of parts when force is applied. The maximum displacement is concentrated in the lower right corner at 4.319 × 10^−5^ mm, and the displacement is very small, which indicates that the arm does not deform when spreading powder. By observing the stress map in Figure 4b, the maximum stress of the powder-spreading arm is concentrated in the middle of the transition part, decreasing toward both ends. The maximum stress is 3.843 × 10^−2^ MPa, which is low. The measured mass of the powder-spreading arm is 2.672 kg. In summary, the stress concentration and deformation of the powder-spreading arm when spreading powder are low, and there is more room for optimization.

### 3.3. Topology Optimization Design of Powder-Spreading Arm for 3D Printer

#### 3.3.1. Topology Optimization Analysis Parameter Settings for Powder-Spreading Arm

**Figure 5:**
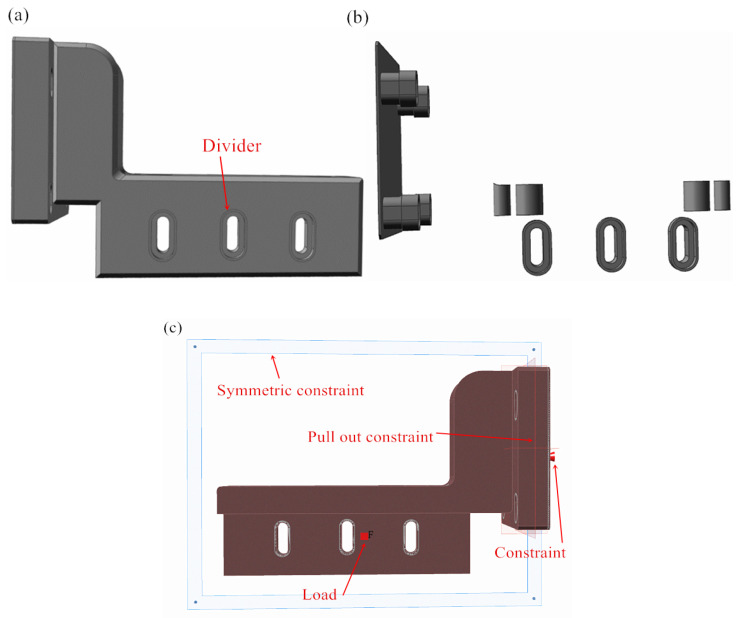
Parameter settings for segmentation and topology optimization analysis of fixed holes in powder-spreading arms: (**a**) segmentation of fixed holes; (**b**) topology optimization analysis parameter settings; (**c**) position of constraints and load application.

In order to ensure that the topology optimization of the powder-spreading arm with its respective screw hole edges and lifting adjustment holes exhibits a complete contour, the screw holes and lifting adjustment holes should be optimized separately. For this reason, we use a split command to separate the screw and lifting adjustment holes, each with a thickness of 1 mm, in the Inspire software. In addition, to prevent the powder from entering the interior of the machine when printing 3D parts, the powder-spreading arm can be divided by 1 mm when in contact with the wall, as shown in Figure 5a. The effect of the screw holes and the division of the wall surface of the powder-spreading arm is shown in Figure 5b. Then, other parts of the powder-spreading arm, excluding the edge of the screw holes and the edge of the elevation adjustment holes, are set as the design space, as shown in the red part in Figure 5. Considering that the powder-spreading arm in contact with the wall is a symmetric structure, the segmentation of the contact area can be set as a symmetric constraint. For the remaining parts of the powder-spreading arm, considering the processing factor, a bidirectional mold-pulling constraint is applied. Setting the same loads and constraints as in Section 3.2, the constraints and loads applied to the powder-spreading arm are shown in Figure 5c. The optimization objective is set to maximize stiffness, and the thickness constraint is 6 mm. To obtain optimal results, the mass objectives are set to 30%, 25%, and 20% for comparative analysis.

#### 3.3.2. Topology Optimization Analysis of the Powder-Spreading Arm

**Figure 6:**
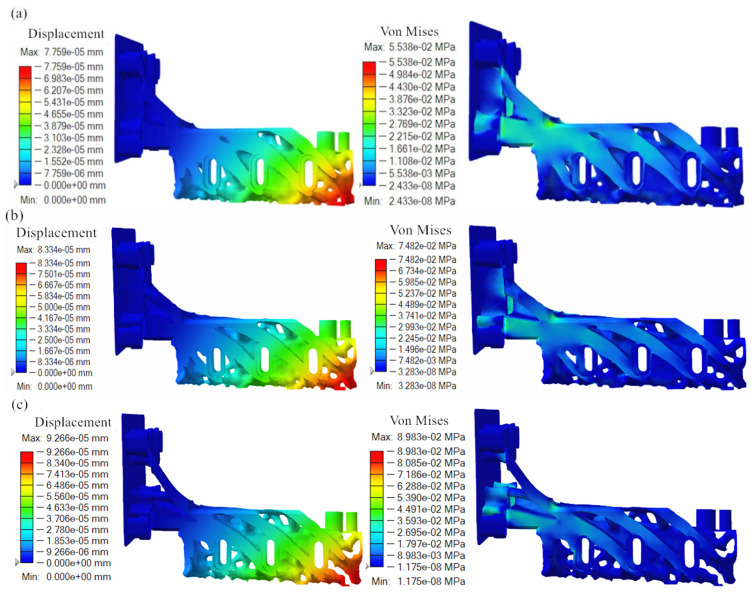
Topology optimization results of powder-spreading arms under different quality objectives: (**a**) 30%; (**b**) 25%; (**c**) 20%.

The topology optimization results of the powder-spreading arm under different mass objectives are shown in Figure 6. Observing the displacement cloud of the powder-spreading arm under different quality objectives, it was found that the displacement of the powder-spreading arm gradually decreases from the lower right corner to the upper left corner, and the changing trend is similar to that before optimization. Comparing the displacement size of the powder-spreading arm under different quality objectives, it was found that the maximum displacement under a 30% quality objective was 7.759 × 10^−5^ mm, and the maximum displacement under a 25% quality objective was 8.334 × 10^−5^ mm. The maximum displacement under a 25% quality objective is 9.266 × 10^−5^ mm. With the increase in the optimization quality objective, displacement increases gradually, which is in line with the law of topology optimization, but the size of the displacement’s throw is smaller. Observing the stress maps of the powder-spreading arm under different quality targets, it was found that the stress distribution of the topology-optimized powder-spreading arm is more uniform from the point of view of stress distribution. A comparison of the stress size of the powder-laying arm under different quality objectives reveals that the maximum stress under a 30% quality objective is 5.538 × 10^2^ MPa, the maximum displacement under a 25% quality objective is 7.482 × 10^−5^ mm, and the maximum displacement under the 25% quality objective is 8.938 × 10^−5^ mm. With an increase in the optimized quality objective, the maximum stress gradually increased, but the increase in throw is smaller. In general, the displacement and maximum stress of the powder-spreading arm under different quality objectives are low, which can meet the requirements of mechanical properties. Combined with the shape of the part after topology optimization, the fracture between the powder-spreading arm and the upper right fixing hole occurs at the 20% quality objective, which is not suitable for being the optimal part, and the powder-spreading arm under the 25% quality objective maintains the integrity of the connection with the fixing hole, simultaneously exhibiting considerable mass reductions. Therefore, we chose the 25% mass objective powder-spreading arm as the optimization result.

### 3.4. Redesign of Powder Arm After Topology Optimization

**Figure 7:**
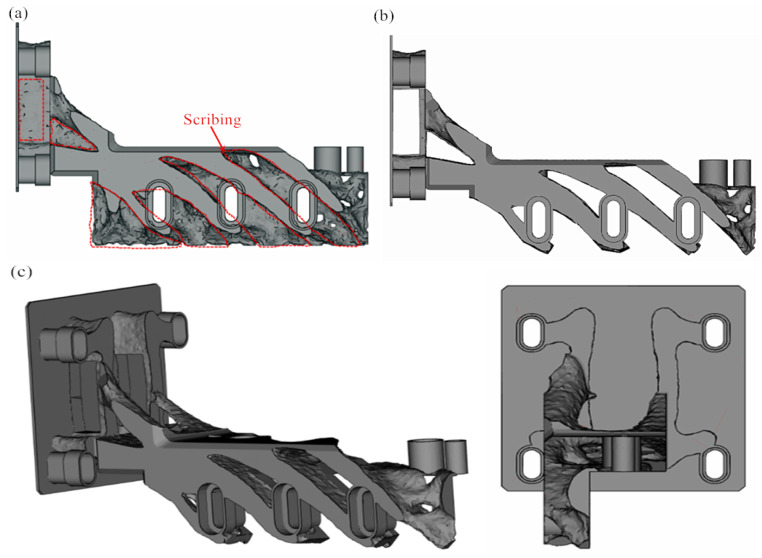
Redesign of powder-spreading arm after topology optimization: (**a**) line cutting; (**b**) the effect after cutting; (**c**) observations from different perspectives.

We further analyzed the structure of the topology-optimized powder-spreading arm and found that the shape of the powder-spreading arm is more complicated, with more lattice-mounted structures and thin-walled parts, which are not conducive to batch processing. For this reason, we saved the topology-optimized powder-laying arm in STL format and imported it into Materialise Magics 22.01 for scribing and cutting, as shown in Figure 7a. The central part of the topology-optimized part can be retained, and the irregular part can be excised during cutting. In addition, due to the considerable strength margin of the topology-optimized part, the thin-walled part is partially excised to further reduce its quality ( Figure 7b). A cross-section of the powder-laying arm is shown from different viewpoints in Figure 7c. Figure 7c reveals that the cross-sectional powder-spreading arm has a clear structure, which can meet mass production requirements due to the substantial reduction in mass. The topology optimization in this paper does not redesign the lifting holes because these are included in the original equipment for compatibility testing.

### 3.5. Three-Dimensional Printing of the Powder-Spreading Arm After Topology Optimization

#### 3.5.1. Data Processing of the 3D-Printed Powder-Spreading Arm

**Figure 8:**
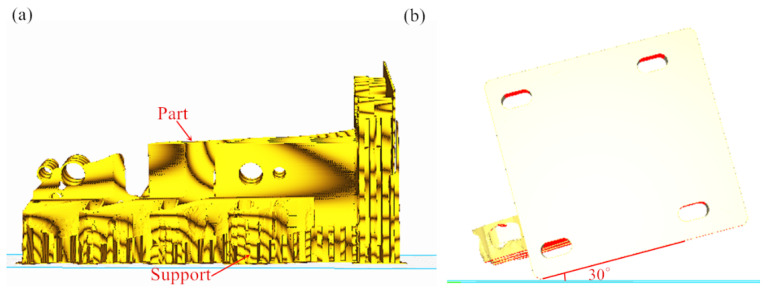
Data processing of 3D-printed powder-spreading arm: (**a**) support addition effect; (**b**) tilted 30° placement method.

The placement and support addition of 3D-printed parts are related to whether the parts can be manufactured successfully. For the powder-laying arm, to avoid adding support to its topology-optimized surface, we adopted a placement with the topology-optimized surface facing upward, as shown in Figure 8a. In addition, in order to prevent the 3D-printed powder-laying arm scan line from being too long, we added support in the holes using a tilted 30° placement, as shown in Figure 8b. Observations from Figure 8 support the addition effect, showing that the powder-laying arm’s topology-optimized surface requires minimal support, with no support needed at the fixed hole, making it easy to remove.

#### 3.5.2. Analysis of the Powder Arm Effect Completed by 3D Printing

**Figure 9:**
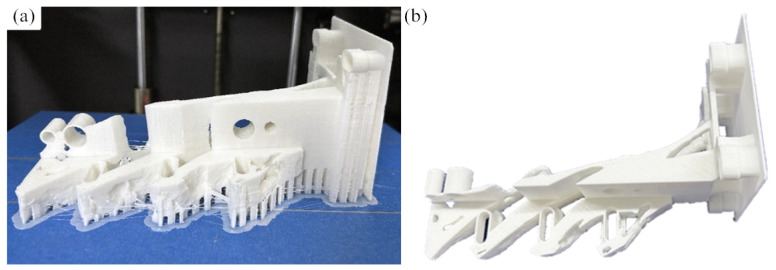
Three-dimensionally printed powder-spreading arm: (**a**) manufactured powder-spreading arm; (**b**) post-processed powder-spreading arm.

The effect of the completed 3D-printed powder-laying arm is shown in Figure 9a. The surface of the printed powder arm parts has a bright, high-quality finish. The hanging surface showed no obvious dregs, and there is no apparent warping or deformation nor any other defects. At the fixed holes and other locations, some support is added, which may affect the surface finish but remains within the permissible range. The finished part is removed from the substrate, and post-processing work, such as removing the support, polishing, sanding, and removing surface burrs, is carried out to obtain the final model, as shown in Figure 9b. Figure 9b shows that the post-processed part has a high-quality surface finish and good structural connections, which can be used for actual assembly inspections.

#### 3.5.3. Matching Inspection of Powder-Spreading Arm Completed by 3D Printing

**Figure 10:**
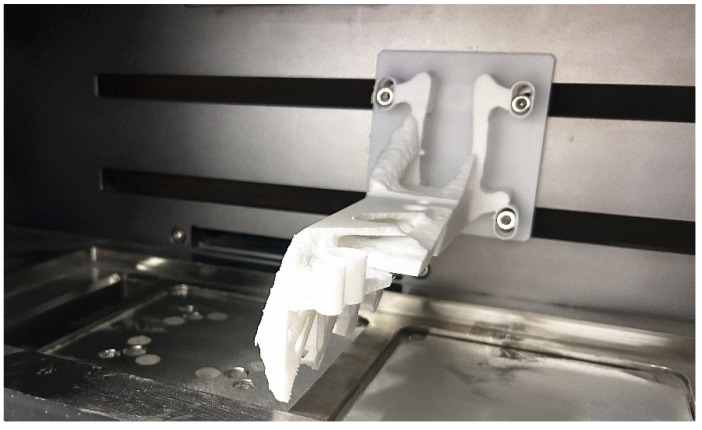
The assembly of the powder-spreading arm completed by 3D printing.

The 3D-printed powder-spreading arm was assembled using screws, and the assembly is shown in Figure 10. As shown in Figure 10, the 3D-printed powder-spreading arm and the assembly wall exhibit close alignment between the screw holes in their proper position. There is no apparent assembly conflict between the components, which indicates that the dimensional accuracy of the completed parts match one another, meeting the subsequent use requirements of metal 3D printers; moreover, these parts can be printed after actual applications.

## 4. Conclusions

(1) The displacement of the powder-spreading arm gradually decreases from the lower right corner to the upper left corner, with the maximum displacement concentrated at the lower right corner measuring 4.319 × 10^−5^ mm. The displacement amount is very small. The maximum stress is concentrated in the middle transition section, decreasing toward both ends, with the maximum stress being 3.843 × 10^−2^ MPa. The stress concentration and deformation during powder-spreading operations carried out by the arm are relatively small, providing ample optimization space.
(2) The topological optimization’s displacement and maximum stress of the powder-spreading arm under different quality targets are relatively small, which can meet mechanical performance requirements. Based on the shape of the part after topological optimization, the powder-spreading arm under the 25% quality target maintains integrity at the fixed hole while achieving a significant reduction in mass.
(3) The 3D-printed powder arm parts have a smooth surface, low roughness, no obvious slag hanging on the overhanging surface, and no obvious warping or deformation. The parts have a high surface finish after post-processing and good structural connections, and they can be used in actual assembly inspections. The fit between the 3D-printed powder arm and the assembly wall is tight, the screw hole positions are appropriate, and there are no apparent assembly conflicts between the components.

Subsequent experiments can improve the arm’s performance, and this lightweight design and 3D printing verification work fills a research gap in lightweight powder-spreading arms, providing technical support for high-performance product design and mass manufacturing. This study is a contribution to improving SLM equipment accuracy, reducing energy consumption, and expanding application scenarios.

## Data Availability

The original contributions presented in this study are included in the article. Further inquiries can be directed to the corresponding author.
